# TLR2 Expression in Relation to IL-6 and IL-1β and their Natural Regulators Production by PMN and PBMC in Patients with Lyme Disease

**DOI:** 10.1155/MI/2006/32071

**Published:** 2006-02-21

**Authors:** Ewa Jablonska, Magdalena Marcinczyk

**Affiliations:** Department of Immunology, Medical University of Bialystok, Wyszynskiego 15A, 15274 Bialystok, Poland

## Abstract

Recently, it has been reported that TLR2 on macrophages plays a unique role in the inflammatory response and host defense to infection with *Borrelia burgdorferi* (*Bb*) which is an etiologic agent of Lyme disease. Experimental studies show that PMNs also play an essential role in infection control by *Bb*. However, there is no available data about TLR2 expression on PMN in the course of Lyme disease. In the present study, TLR2 expression and production of IL-1β and IL-6 as well as their natural regulators (sIL-1RII, IL-1Ra and sIL-6Rα, sgp130, resp) by PMN of peripheral blood in patients with Lyme disease were examined. For the purpose of comparison, the same activity of autologous peripheral blood mononuclear cells (PBMCs) was estimated. An effect of rhIL-15 on TLR2 and cytokine secretion was also studied. Increased TLR2 expression in unstimulated neutrophils suggests an important role of these cells in mechanism recognition of *B burgdorferi* in patients with Lyme disease. The relationship between IL-1β and IL-6 as well as their regulators by unstimulated PMN and PBMC, observed in the present study, may lead to enhanced IL-6- and to inhibition of IL-1β-mediated reactions in this patient group. Changes in the TLR2 expression after rhIL-15 stimulation appear to have a favorable effect on mechanism recognition of *Bb*. The relations between IL-6 and its regulators (sIL-6Rα and sgp130) as well as between IL-1β and its regulators (IL-1Ra and sIL-1RII) after rhIL-15 stimulation may lead to enhanced IL-1β- and IL-6-mediated inflammatory reactions in the course of Lyme disease.

## INTRODUCTION

Toll-like receptors (TLRs) are important pattern-recognition receptors that play an essential role in activating signal transduction pathways leading to killing and clearance of pathogens. TLRs recognize highly conserved, pathogen-coded molecular structures termed pathogen-associated molecular patterns (PAMPs) [1, 2]. TLRs are members of the IL-1R superfamily. In cytoplasmic domain, TLRs show significant homology to the IL-1 receptor type I and IL-18 receptor. This domain contains a conserved region called the Toll/IL-1R (TIR). Recognition of PAMPs by TLRs recruits MyD88, IRAK kinase, and TRAF6 [1]. These adaptor molecules mediate the activation of the JNK, p38, ERK 1/2, NF-κB, and phosphoinositide 3-kinase signaling pathways leading to activation of inflammatory target genes [3].

Eleven human TLRs have been identified: TLR1, TLR2, TLR3, TLR4, TLR5, TLR6, TLR7, TLR8, TLR9, TLR10, and TLR11. They are expressed in a wide range of human innate as well as in adaptive cells [1, 4–6].

Human polymorphonuclear neutrophils (PMNs), the primary effector cells in the first line of defense infection factors, express nine of all identified TLRs except for TLR3 [4, 7]. Among the extensively characterized receptors, TLR2 is crucial for the inflammatory response to components of gram-positive and gram-negative bacteria and mycobacteria such as PGN, lipoteichoic acid, bacterial lipoproteins, lipopeptides, and lipoarabinomannan [7–9].

Recently, it has been reported that TLR2 on macrophages mediates responses to lipoproteins of *Borrelia burgdorferi*, which is an etiologic agent of Lyme disease. Experimental studies show that PMNs play a critical role in infection control by *Bb* through their phagocytosis both in presence and in absence of specific antibody [10]. However, there is no available data about TLR2 expression in PMN of patients with Lyme disease. It was well established that PMN besides phagocytosis can synthesize and release a wide range of inflammatory cytokines such as IL-1β, IL-6, IL-8, IL-15, or TNF-α [11–13]. Available data indicates that activation of TLRs can trigger the release of cytokines and chemokines [7].

It has been reported that PMNs have also ability to release cytokine-regulatory proteins that may control the activity of certain cytokines in biological fluids or in tissue culture. PMNs are capable of producing soluble IL-1 receptor type II (sIL-1RII) that retains its affinity for IL-1 ligand but does not transduce the signal and thus is regarded as an IL-1 inhibitor [14]. Human PMN can also generate receptor-binding antagonists, such as IL-1Ra that competitively inhibits binding of IL-1β to IL-1 receptors without exhibiting detectable agonist activity [14]. Furthermore, it was well documented that PMNs release IL-6 regulators: soluble IL-6 receptor α (sIL-6R α) that acts as IL-6 agonist and soluble gp130 (sgp130) that acts as IL-6 antagonist [15].

The ability of PMN to release cytokines and their natural regulators is controlled by a wide range of other cytokines [13–16]. It was well established that IL-15 plays an important role in the innate immunity and potentiates several functions of normal neutrophils [13, 17]. Our previous study indicated the modulatory effect of rhIL-15 on the PMN chemotaxis and phagocytosis in patients with Lyme disease [12]. Moreover, during other earlier examinations, we showed an IL-15 influence on IL-1β and IL-1Ra secretion by PMN from healthy subjects [17].

In the present study, TLR2 expression in relation to expression and production of IL-1β, IL-6, as well as their natural regulators (sIL-1RII, IL-1Ra and sIL-6Rα, sgp130, resp) production by PMN of peripheral blood in patients with Lyme disease were estimated. For the purpose of comparison, the same activity of autologous peripheral blood mononuclear cells (PBMCs) was studied. RhIL-15 effect on TLR2 expression and production of cytokines was also examined. TLR2 expression in relation to proinflammatory cytokines may explain its role in the maintenance of balance between these mediators. Furthermore, relations between these molecules after rhIL-15 stimulation may provide new data on this cytokine disease.

## MATERIAL AND METHODS

### Patients

A total of 43 patients diagnosed with Lyme borreliosis and treated in the Department of Parasitic Diseases and Neuroinfections, Medical University of Bialystok, were enrolled into the study. There were 28 males and 12 females aged from 32 to 55 years. Erythema migrans was found in 2 patients, neuroboreliosis in 8, Lyme arthritis in 19, and boreliosis in 14. The diagnosis was established based on serologic tests for IgM and IgG antibodies to *Borrelia burgdorferi* flagellum antigen.

Most of patients had increased leukocytosis. The mean number of white cells in blood was 7.02^3^/μl (from 3.8 to 24.75^3^/μl). The mean percent of PMN in the peripheral blood of patients was 58.9% (from 34 to 73%).

Blood samples were taken from each patient before treatment.

#### Control

Control subjects (*n* = 30) were healthy people aged from 32 to 53 years.

### PMN culture

Cells were isolated from heparinized (10 U/mL) whole blood by Gradisol G gradient 1.115 g/mL (Polfa) by Zeman et al. This method enables simultaneous separation of two highly purified leukocyte fractions: mononuclear cells (PBMCs), containing 95% lymphocytes, and polymorphonuclear cells containing 94% neutrophils (PMNs). The purity of isolated PMNs was determined by May-Grunewald-Giemsa staining. The cells were suspended in the culture medium (RPMI-1640, autologous serum, penicillin, and streptomycin) to provide 5 × 10^6^ cells/mL and the cells were incubated in flat-bottomed 96-well plates (Microtest III-Falcon) for 18 h at 37°C in a humidified incubator with 5% CO_2_ (NuAire Systems, Inc, Tenn, USA). LPS (10 μg/mL; Difco, Detroit, Mich, USA) was tested to stimulate secretion by PMN. RhIL-15 (50 ng/mL; R & D Systems) was tested to stimulate secretion by PMN and PBMC. After culture, the viability of the PMN was > 90%, PBMC > 92% as determined by trypan blue exclusion.

After 18 h incubation, supernatants were removed and assessed for IL-1β, IL-6, sIL-1RII, IL-1Ra, sIL-6Ra, and sgp130 using ELISA kit (BioSource International, Inc, Camarillo, USA).

### Western blot analysis

Cytoplasmic protein fractions of PMN and PBMC were analyzed for the presence of TLR2, IL-1β, sIL-1RII, IL-1Ra and IL-6, sIL-6Ra, sgp130 by western blotting. Cells were lysed directly in the presence of inhibitor protease (Sigma) by sonication using Vibra-Cell Ultrasonic Processor (Sonics & Materials, Inc, USA). Protein fractions were suspended in Laemmli buffer (Bio-Rad Laboratories, Herkules, Calif, USA) and then were electrophorezed on SDS-PAGE. The resolved protein was transferred onto 0.2 μm pore-sized nitrocellulose (Bio-Rad Laboratories, Herkules, Calif, USA). The nitrocellulose was incubated at +4°C for 18 h with the primary monoclonal antibody anti-TLR2 (Alexis, Carlsbad, Calif, USA), anti-IL-1β, anti-sIL-1RII, anti-IL-1Ra, anti-IL-6, anti-sIL-6Ra, and anti-sgp130 (R & D Systems, Minneapolis, USA). After washing with 0.1% TBS-T, the membrane was incubated at room temperature for 1 h with alkaline phosphatase antimouse IgG Abs (Vector Laboratories, Burlingame, Calif, USA). Immunoreactive protein bands were visualized following the addition of AP-Conjugate Substrate Kit (Bio-Rad Laboratories, Herkules, Calif, USA). Band intensity was quantified using LabImage 1 D gel software.

### IL-1β, IL-6, sIL-1RII, IL-1Ra, sIL-6Ra, and sgp130 in the serum

 IL-1β, IL-6, sIL-1RII, IL-1Ra, sIL-6Ra, and sgp130 levels in the serum were assessed using ELISA kit (BioSource International, Inc, Camarillo, USA).

### Statistical analysis

The results including cytokines are expressed as mean + standard deviation. Data was analyzed according to the nonparametric U Mann-Whitney test. Correlations were calculated using Pearson's test. A *P* value less than .05 was considered statistically significant.

## RESULTS

### Western blot analysis

Western blot analysis showed that the samples of unstimulated PMN and PBMC contained a 86 kd protein that was stained by an anti-TLR2 monoclonal antibody (Figure 1(A,A′)). These samples also contained 17kd protein stained by an anti-IL-1β (Figure 1(B,B′)), 45 kd protein stained by an anti-sIL-1RII (Figure 1(C,C′)), 18 kd protein stained by an anti-IL-1Ra (Figure 1(D,D′)), 26 kd protein stained by an anti-IL-6 (Figure 1(E,E′)), 55 kd protein stained by an anti-sIL-6Rα (Figure 1(F,F′)), and 90–110 kd protein stained by an anti-sgp130 (Figure 1(G,G′)).

The rhIL-15-stimulated PMN and PBMC of patients cells expressed an increase of TLR2 protein in comparison with the unstimulated cells (Figure 1(A,A′)). Incubated PMN and PBMC of patients with rhIL-15 showed higer expression of IL-1β compared to unstimulated cells (Figure 1(B,B′)). In contrast to PBMC, sIL-1RII expression in PMN of patients was on the same level as in unstimulated cells in this group (Figure 1(C,C′)). The bands of IL-1Ra and IL-6 in rhIL-15-stimulated PBMC and PMN of patients were at the same level as in unstimulated cells too (Figure 1(D,D′,E′)). Expression of IL-6 and sIL-6Rα in PMN was higher in comparison to unstimulated cells in patients group (Figure 1(E,F)). There were not any differences between expression of sgp130 in PMN and PBMC from patients between unstimulated and stimulated with rhIL-15 (Figure 1(G,G′)).

### Cytokines concentrations in the culture supernatants of PMN and PBMC

In the culture supernatants of PMN and PBMC, we did not find differences in the concentrations of IL-1β and IL-1Ra between patients and the control group (Table 1). In both groups, stimulation with rhIL-15 or LPS induced significant increase in IL-1β and IL-1Ra release. Concentrations of IL-1β in culture supernatants of stimulated PMN of patients were statistically lower than those in cultures of the control group. We also found higher concentrations of IL-1β in culture supernatants of PBMC as compared to PMN of control and patient groups (Table 1). In contrast, unstimulated PMN and unstimulated and stimulated PBMC from patients with Lyme disease secreted higher concentrations of sIL-1RII than the cells from healthy subjects (Table 1). The release of sIL-1RII was not significantly different in cultures of unstimulated and stimulated PMN and PBMC in patients.

Unstimulated and rhIL-15-stimulated PMN and PBMC from patients with Lyme disease secreted higher concentrations of IL-6 than cells from healthy control (Table 2). The secretion of sIL-6Rα by unstimulated PMN and PBMC was not significantly different between patient and control groups (Table 2). PMN and PBMC from patients treated with rhIL-15 produced higher amounts of sIL-6Rα than untreated cells. The mean value of sgp130 in culture supernatants of unstimulated and stimulated PMN and PBMC was not significantly different between patient and control groups.

### Cytokines concentrations in the serum

The concentrations of IL-1β, IL-1Ra, IL-6, sIL-6Rα, sgp130, and IL-15 in the serum of patients with Lyme disease were significantly higher than those in the serum of control group (Table 3).

### Relationships between estimated cytokines in culture supernatants of cells and the serum

Although there was no correlation between IL-1β, IL-1Ra, IL-6 and their receptors release by PMN, PBMC and the serum levels in patients with Lyme disease, a coincidence between them was found. This appears to suggest that other cells are also responsible for their concentrations in sera of this patient group. In contrast, a direct correlation between the release of sIL-1RII by PBMC and sera levels was shown indicating that these cells are main source of circulating sIL-1RII (*r* = 0.63, *P* < .05).

## DISCUSSION

In the present study, changes in TLR2 expression and cytokine production in freshly isolated PMN and PBMC from patients with Lyme disease before treatment were found.

Increased expression of TLR2 in PMN and PBMC may lead to enhance the mechanism recognition of spirochetes *B burgdorferi* and their elimination. Significant role of TLR2 in the control of *B burgdorferi* infection was confirmed by a fact that deficiency in TLR2 greatly impaired the host defense to *Bb*, resulting in more number of spirochetes in tissues [18].

High expression of TLR2 in PMN and PBMC from patients may be caused by outer surface lipoproteins of *B burgdorferi* such as OspA or OspB *in vivo* [8, 19]. It has been demonstrated that TLR2-expressing macrophages are stimulated by *Bb* suggesting that TLR2 recognition of lipoproteins is relevant to natural Borrelia infection [19]. Results of Bulut et al have shown that *Bb* outer surface lipoprotein A (OspA-L) signals through TLR2 [8]. They also found that OspA-L requires functional cooperation of TLR6 and TLR2 to signal transduction. This combination of TLRs facilitates mammalian responsiveness to a wide array of pathogen-associated molecular patterns and diversifies the repertoire of Toll-mediated responses [20].

Despite the increase in TLR2 expression, elevated IL-6 production by examined leukocytes was also observed. Simultaneous enhanced TLR2 expression and IL-6 production by examined cells suggest that TLR2 engagement can up-regulate neutrophil inflammatory cytokine production. This is in agreement with data, indicating that TLR2 signal triggers expression of inflammatory cytokines, involving IL-6 [21].

Similar to TLR2 are lipoproteins of *B burgdorferi* that possess cytokine-stimulating properties and may be responsible for enhanced secretion of IL-6 by PMN and PBMC of patients with Lyme diseases [16]. Increased IL-6 production by these cells from patients may enhance the inflammatory reactions leading to *B burgdorferi* elimination. On the other hand, elevated IL-6 activity can make a contribution to long-lasting inflammatory process in patients with Lyme disease especially that its inhibitor—sgp130—release was unchanged.

In contrast to IL-6 family proteins, unchanged production of IL-1β was associated with high secretion of its inhibitor—sIL-1RII by PMN and PBMC of patient group. Lack of significant alteration in secretion of IL-1β may be caused by a suppressive effect of IL-6. In contrast, IL-1β has ability to enhance the IL-6 production by immune cells [22]. Furthermore, increased sIL-1RII production by these cells, acting as inhibitor for IL-1β, may lead to decrease its activity in patient with Lyme disease. Thus, unchanged secretion of IL-1β together with increased secretion of sIL-1RII by PMN may be a protect mechanism of host against excessive activity of IL-6 in early phase of inflammation. In contrast, the studies curried out by Brown et al suggest that sIL-1RII plays an important role in the late inflammation, specifically working as a reservoir for remaining IL-1β and preventing the change of extracellular pro-IL-1β into active IL-1β [23]. Furthermore, unchanged IL-1Ra secretion by PMN and PBMC can be one of the host defense mechanism to inflammatory process. Several studies showed that IL-1Ra reduced markedly the infiltration of inflammatory cells to tissue sites [24]. Moreover, IL-1Ra can inhibit IL-8 secretion by PBMC induced by IL-18 [25]. Interesting data including IL-1β and IL-1Ra in patients with Lyme disease by Miller et al was found [26]. They suggested that relationship between these proteins influences progression of Lyme arthritis. However, we did not find distinct relations between IL1β and IL-1Ra in patients with Lyme arthritis in comparison to patients with neuroborreliosis, borreliosis, or erythema migrans.

 Increased TLR2 and cytokines expression can be controlled by other cytokines. For example, it was well established that TLR2 on monocytes is upregulated by IL-1 [27].

A new role of IL-15 in the regulation of human TLR2 expression was shown for the first time, by our results showing an effect of rhIL-15 on its increased expression in PMN and PBMC *in vitro*. This data is similar to results of Musikacharoen et al who reported that mouse TLR2 mRNA was rapidly induced by IL-15 [27].

It is interesting to note increased secretion of IL-6 and its agonist sIL-6Ra by PMN and PBMC after rhIL-15 stimulation leading to enhance IL-6-mediated response in patients with Lyme disease. In contrast, increased production of IL-1β by cells stimulated with rhIL-15 is balanced by simultaneous increased secretion of its antagonist—IL-1Ra that can block IL-1β-mediated reactions.

 Concluding, increased TLR2 expression in neutrophils of patients with Lyme disease indicates an important role of these cells in recognition of *B burgdorferi*. TLR2 expression in relation to expression and production of cytokines and their regulators suggests a role of this receptor in enhanced IL-6- and inhibition of IL-1β-mediated reactions in patients with Lyme disease. Changes in the TLR2 expression after rhIL-15 stimulation suggest a favorable effect of this cytokine on mechanism recognition of *Bb* by PMN and PBMC. In contrast, changes in the release of IL-6 and sIL-6Ra after rhIL-15 stimulation suggest an unfavorable effect of this cytokine leading to enhance IL-6 activity in the course of Lyme disease. Observations above clearly suggest that further examinations are needed to explain an actual role of IL-15 in immune and inflammatory response during infection control by *B burgdorferi*.

## Figures and Tables

**Figuer 1:**
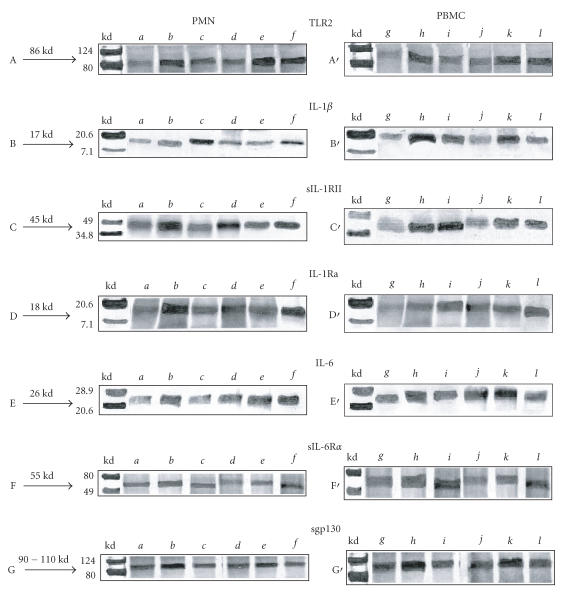
Western blot analysis of TLR2 and IL-1β, sIL-1RII, IL-1Ra, IL-6, sIL-6Rα, sgp130 in human PMN and PBMC cells from control and patients with Lyme disease. *a*: PMN of control. *b*: PMN+LPS of control. *c*: PMN+rhIL-15 of control. *d*: PMN of patients. *e*: PMN+LPS of patients. *f*: PMN+rhIL-15 of patients. *g*: PBMC of control. *h*: PBMC+LPS of control. *i*: PBMC+rhIL-15 of control. *j*: PBMC of patients. *k*: PBMC+LPS of patients. *l*: PBMC+rhIL-15 of patients.

**Table 1:** IL-1β, sIL-1RII, and IL-1Ra in the culture supernatants of PMN and PBMC from control and patients with Lyme disease.

	Control *n* = 30		Patients *n* = 43	

	PMN	PBMC	PMN	PBMC
	$\bar{x}$ ± SD	$\bar{x}$ ± SD	$\bar{x}$ ± SD	$\bar{x}$ ± SD

IL-1β (pg/mL)				

Unstimulated cells	7 ± 3.7	13 ± 7^b^	8.8 ± 2.8	18 ± 3.9^b^
LPS-stimulated	22 ± 7.6^a^	46 ± 13^a^ ^b^	13 ± 4.9^a^ ^*^	39 ± 12^a^ ^b^
rhIL-15 stimulated	28 ± 10^a^	40 ± 14^a^ ^b^	11 ± 3^a^ *	32 ± 10^a^ ^b^

sIL-1RII (ng/mL)				

Unstimulated cells	0.6 ± 0.2	0.6 ± 0.2	0.9 ± 0.5*	1 ± 0.4^b^ *
LPS-stimulated	1 ± 0.2^a^	1 ± 0.3^a^	1 ± 0.5	1.7 ± 0.4^b^ *
rhIL-15 stimulated	0.9 ± 0.3^a^	0.9 ± 0.2^a^	1 ± 0.5	1.6 ± 0.5^b^ *

IL-1Ra (ng/mL)				

Unstimulated cells	0.6 ± 0.2	0.5 ± 0.2	0.6 ± 0.3	0.4 ± 0.2
LPS-stimulated	1 ± 0.4^a^	1 ± 0.4^a^	0.8 ± 0.3 ^a^	1 ± 0.7^a^
rhIL-15 stimulated	1 ± 0.3^a^	1 ± 0.3^a^	1.4 ± 0.4^a^	1.8 ± 0.9^a^

*Statistical differences between control and patients groups (*P* < .001).

^a^Statistical differences between unstimulated cells and cells incubated with LPS or rhIL-15 (*P* < .001).

^b^Statistical differences between PMN and PBMC (*P* < .001).

**Table 2:** IL-6, sIL-6Rα, and sgp130 in the culture supernatants of PMN and PBMC from control and patients with Lyme disease.

	Control *n* = 30		Patients *n* = 43	

	PMN	PBMC	PMN	PBMC
	$\bar{x}$ ± SD	$\bar{x}$ ± SD	$\bar{x}$ ± SD	$\bar{x}$ ± SD

IL-6 (pg/mL)				

Unstimulated cells	8 ± 3.3	24 ± 4.7^b^	19 ± 8.3*	39 ± 14^b^ *
LPS-stimulated	16 ± 3.8^a^	39 ± 9.0^a^ ^b^	21 ± 6.5	43 ± 13^b^
rhIL-15 stimulated	11 ± 2.7^a^	29 ± 6.1^b^	26 ± 9.3^a^ *	42 ± 12^b^ *

sIL-6Rα (ng/mL)				

Unstimulated cells	2.3 ± 0.9	4 ± 1.4^b^	1.6 ± 0.9	3 ± 0.9^b^
LPS-stimulated	3.9 ± 1.0^a^	5.4 ± 2.1	2.2 ± 0.8^a^ *	3.5 ± 0.9^b^ *
rhIL-15 stimulated	3 ± 0.8	5.2 ± 1.9^b^	2 ± 0.5^a^	3.4 ± 0.6^a^ ^b^ *

sgp130 (ng/mL)				

Unstimulated cells	29 ± 9.2	31 ± 6.4	28 ± 6.7	27 ± 8.4*
LPS-stimulated	42 ± 10^a^	44 ± 12^a^	37 ± 10^a^	37 ± 12^a^
rhIL-15 stimulated	33 ± 8.7	36 ± 6.7	30 ± 8.2	33 ± 7.3*

*Statistical differences between control and patients groups (*P* < .001).

^a^Statistical differences between unstimulated cells and cells incubated with LPS or rhIL-15 (*P* < .001).

^b^Statistical differences between PMN and PBMC (*P* < .001).

**Table 3:** The mean concentrations of IL-1β, sIL-1RII, IL-1Ra, IL-6, sIL-6Rα, and sgp130 in the serum of control and patients with Lyme disease.

	Serum	
	Control *n* = 30	Patients *n* = 43
	$\bar{x}$ ± SD	$\bar{x}$ ± SD

IL-1β (pg/mL)	3.7 ± 1.4	36 ± 10*
sIL-1RII (ng/mL)	4.1 ± 1.6	4.3 ± 1.2
IL-1Ra (ng/mL)	0.4 ± 0.1	0.8 ± 0.3*
IL-6 (pg/mL)	5.3 ± 0.9	12 ± 5.5*
sIL-6R (ng/mL)	14 ± 6.7	56 ± 5.1*
sgp130 (ng/mL)	37 ± 5.9	72 ± 8.1*
IL-15 (pg/mL)	290 ± 91	383 ± 101*

*Statistical differences between control and patients groups (*P* < .001).

## References

[1] Doyle SE, O'Connell RM, Miranda GA (2004). Toll-like receptors induce a phagocytic gene program through p38. *The Journal of Experimental Medicine*.

[2] Sabroe I, Read RC, Whyte MKB, Dockrell DH, Vogel SN, Dower SK (2003). Toll-like receptors in health and disease: complex questions remain. *The Journal of Immunology*.

[3] Takeuchi O, Akira S (2002). Genetic approaches to the study of Toll-like receptor function. *Microbes and Infection*.

[4] Sabroe I, Prince LR, Jones EC (2003). Selective roles for Toll-like receptor (TLR)2 and TLR4 in the regulation of neutrophil activation and life span. *The Journal of Immunology*.

[5] Flo TH, Halaas Ø, Torp S (2001). Differential expression of Toll-like receptor 2 in human cells. *Journal of Leukocyte Biology*.

[6] Zhang D, Zhang G, Hayden MS (2004). A Toll-like receptor that prevents infection by uropathogenic bacteria. *Science*.

[7] Hayashi F, Means TK, Luster AD (2003). Toll-like receptors stimulate human neutrophil function. *Blood*.

[8] Bulut Y, Faure E, Thomas L, Equils O, Arditi M (2001). Cooperation of Toll-like receptor 2 and 6 for cellular activation by soluble tuberculosis factor and *Borrelia burgdorferi* outer surface protein A lipoprotein: role of Toll-interacting protein and IL-1 receptor signaling molecules in Toll-like receptor 2 signaling. *The Journal of Immunology*.

[9] Lien E, Sellati TJ, Yoshimura A (1999). Toll-like receptor 2 functions as a pattern recognition receptor for diverse bacterial products. *The Journal of Biological Chemistry*.

[10] Lusitani D, Malawista SE, Montgomery RR (2002). *Borrelia burgdorferi* are susceptible to killing by a variety of human polymorphonuclear leukocyte components. *The Journal of Infectious Diseases*.

[11] Cassatella MA (1995). The production of cytokines by polymorphonuclear neutrophils. *Immunology Today*.

[12] Jablonska E, Marcinczyk M, Izycka A, Hermanowska-Szpakowicz T (2003). Effect of interleukin 15 on the PMN activity in Lyme borreliosis [in Polish]. *Polski Merkuriusz Lekarski*.

[13] McDonald PP, Russo MP, Ferrini S, Cassatella MA (1998). Interleukin-15 (IL-15) induces NF-κB activation and IL-8 production in human neutrophils. *Blood*.

[14] Roux-Lombard P (1998). The interleukin-1 family. *European Cytokine Network*.

[15] Jablonska E, Marcinczyk M (2003). Role of interleukin-15 and interleukin-18 in the secretion of sIL-6R and sgp130 by human neutrophils. *Mediators of Inflammation*.

[16] Jabłońska E, Marcińczyk M, Talarek Ł, Pancewicz S, Hermanowska-Szpakowicz T, Jabłoński J (2003). IL-15 in the culture supernatants of PMN and PBMC and the serum of patients with Lyme disease. *Roczniki Akademii Medycznej w Białymstoku*.

[17] Girard D, Paquet ME, Paquin R, Beaulieu AD (1996). Differential effects of interleukin-15 (IL-15) and IL-2 on human neutrophils: modulation of
phagocytosis, cytoskeleton rearrangement, gene expression, and apoptosis by IL-15. *Blood*.

[18] Wooten RM, Ma Y, Yoder RA (2002). Toll-like receptor 2 is required for innate, but not acquired, host defense to *Borrelia burgdorferi*. *The Journal of Immunology*.

[19] Giambartolomei GH, Dennis VA, Lasater BL, Philipp MT (1999). Induction of pro- and anti-inflammatory cytokines by *Borrelia burgdorferi* lipoproteins
in monocytes is mediated by CD14. *Infection and Immunity*.

[20] Häupl T, Landgraf S, Netusil P (1997). Activation of monocytes by three OspA vaccine candidates: lipoprotein OspA is a potent stimulator of monokines. *FEMS Immunology and Medical Microbiology*.

[21] Barton GM, Medzhitov R (2002). Control of adaptive immune responses by Toll-like receptors. *Current Opinion in Immunology*.

[22] Schindler R, Mancilla J, Endres S, Ghorbani R, Clark SC, Dinarello CA (1990). Correlations and interactions in the production of interleukin-6 (IL-6), IL-1, and tumor necrosis factor (TNF) in human
blood mononuclear cells: IL-6 suppresses IL-1 and TNF. *Blood*.

[23] Brown EA, Dare HA, Marsh CB, Wewers MD (1996). The combination of endotoxin and dexamethasone induces type II interleukin 1 receptor (IL-1r II) in monocytes: a comparison
to interleukin 1 β (IL-1 β) and interleukin 1 receptor antagonist (IL-1ra). *Cytokine*.

[24] DeForge LE, Tracey DE, Kenney JS, Remick DG (1992). Interleukin-1 receptor antagonist protein inhibits interleukin-8 expression in lipopolysaccharide-stimulated human whole blood. *The American Journal of Pathology*.

[25] Puren AJ, Razeghi P, Fantuzzi G, Dinarello CA (1998). Interleukin-18 enhances lipopolysaccharide-induced interferon-γ production in human whole blood cultures. *The Journal of Infectious Diseases*.

[26] Miller LC, Isa S, Vannier E, Georgilis K, Steere AC, Dinarello CA (1992). Live Borrelia burgdorferi preferentially activate interleukin-1 beta gene expression
and protein synthesis over the interleukin-1 receptor antagonist. *The Journal of Clinical Investigation*.

[27] Musikacharoen T, Matsuguchi T, Kikuchi T, Yoshikai Y (2001). NF-κ B and STAT5 play important roles in the regulation of mouse Toll-like receptor 2 gene expression. *The Journal of Immunology*.

